# Familiarity between patient and general practitioner does not influence the content of the consultation

**DOI:** 10.1186/1471-2296-9-51

**Published:** 2008-09-24

**Authors:** Lea Jabaaij, Thijs Fassaert, Sandra van Dulmen, Arno Timmermans, Gerrit A van Essen, François Schellevis

**Affiliations:** 1 NIVEL (Netherlands Institute for Health Services Research), P.O. Box 1568, 3500 BN Utrecht, the Netherlands; 2 NHG (Dutch College of General Practitioners), Utrecht, the Netherlands; 3 Julius Center for Health Sciences and Primary Care, University Medical Center Utrecht, The Netherlands; 4 Department of General Practice, VU University Medical Centre, Amsterdam, the Netherlands

## Abstract

**Background:**

Personal continuity in general practice is considered to be a prerequisite of high quality patient care based on shared knowledge and mutual understanding. Not much is known about how personal continuity is reflected in the content of GP – patient communication. We explored whether personal continuity of care influences the content of communication during the consultation.

**Methods:**

Personal continuity was defined as the degree of familiarity between GP and patient, rated by both the GP and the patient. 394 videotaped consultations between GPs and patients aged 18 years and older were analyzed. GP – patient communication was evaluated with an observation checklist, which rated the following topics of conversation: (1) medical issues, (2) psychological themes, and (3) the social environment of the patient. For each of these topics we coded whether or not it received attention, and was built upon prior knowledge. Data were analyzed using multilevel logistic regression analyses.

**Results:**

No relationship was found between GP – patient familiarity and the discussion of medical issues, psychological themes, or the social environment of the patient. But if the patient and the GP knew each other very well, the GP more often displayed prior knowledge with the topic in question. Few patient and GP characteristics were associated with differences in content of communication.

**Conclusion:**

Given the relatively small sample size, we carefully conclude that familiarity between a GP and a patient does not influence the content of the communication (medical issues, psychological themes nor topics relating to the social environment). This is remarkable because we expected that familiarity would 'open up the communication' for more psychological and social themes. GPs seem to have the communication skills to put both familiar and non-familiar patients at ease enabling them to freely raise any issue they think necessary.

## Background

Traditionally, continuity of care (COC) is a "core value" to primary health care, internationally, as well as in Dutch General Practice. Already in the fifties, the Dutch College of General Practitioners defined COC as continuous, integral and personal healthcare by general practitioners (GPs) for patients or families registered in their practice. This classic definition of COC refers mainly to what is now called (inter)personal continuity, i.e. the ongoing relationship between an individual patient and an individual GP [1]. Several studies report that the use of health care resources is influenced by the degree of familiarity between GP and patient. It saves time when the GP knows the patient and this is helpful in deciding on diagnostic activities and therapeutic actions [2,3]. On the other hand, recently enlisted patients who did not yet have an established relationship with their new GP, have a higher chance of having a contact with the GP, receiving a prescription or a referral [4]. Having a long-lasting relationship with a GP is also related to improved preventive care, reduced hospitalization [5] and lower total health care costs [6].

Personal continuity or familiarity between a patient and a GP has also been associated with favourable patient outcomes. For example, sustained physician-patient relationships with mutual trust and GPs' knowledge of patients have been found to be positively associated with patient enablement [7], medication compliance [8], patient satisfaction [9], adherence to physician's advice, and improved health status [10].

Interpersonal communication is essential in creating and maintaining good interpersonal relationships between doctors and patients [11-13]. Patients report better communication with a personal GP [13,14]. This relationship is reciprocal, as good interpersonal communication is thought to stimulate the experience of personal care and is necessary to build a good GP – patient relationship. Not surprisingly, personal continuity and effective GP – patient communication are thought to be especially relevant to patients who have psychological, emotionally laden or more serious problems [15,16].

Continuous patient care by the same GP is increasingly difficult to achieve, as personal continuity of care is threatened by factors like expanding practice size and a growing number of GPs working part-time. At the same time, several studies have suggested that the extent to which patient and GP know each other might be more important than merely seeing the same doctor every time [7,17]. The concept of knowing a doctor well is complex and research is needed to determine whether knowing the doctor is a suitable proxy-measure for personal continuity [7]. Although it seems obvious that personal continuity is reflected in GP – patient communication, this relationship has not been studied very well. It is unclear whether more personal continuity elicits different conversations, or whether it is associated with the use of prior knowledge.

This study explores the relation between personal continuity, in terms of the extent to which GP and patient know each other, and the content of GP – patient communication during consultations. The topics we have focused on relate to medical issues, psychological themes, and the social environment of the patient. For each of these topics it was measured whether they were discussed, and whether the GP displayed prior knowledge. It was hypothesized that the more GPs and patients were familiar with each other, the more GPs would (a) discuss non-medical issues, and (b) explicitly integrate prior knowledge in their consultations.

## Methods

### Design

This study was carried out using systematic observations of 400 videotaped GP patient contacts of 40 GPs. The relationship between familiarity and content of communication was studied in multilevel logistic regression analyses. Several patient and GP factors were tested for their possibly explanatory role.

The videotaped consultations were selected from a larger data set of 2368 consultations. We will first describe the data available from this larger data set, after which we describe how the sample for the present study was selected.

### Initial data collection

#### Setting and sample characteristics

The study was conducted with data from the second Dutch National Survey of General Practice (DNSGP-2), carried out by NIVEL in 2001 [18]. As part of this survey, consultations of 142 GPs (108 men and 34 women) were videotaped [19]. Fifteen to twenty consultations per GP were videotaped, resulting in a database of 2368 consultations with patients aged 18 years and older.

#### Patients

On a random day, consecutive patients consulting the GP's office were approached and informed about the study. They were not informed beforehand. All patients were asked permission to videotape their consultation. If they agreed, they signed a consent form. The privacy of the collected data was laid down in privacy regulations, by which ethical consent was safeguarded.

Patient information was derived from electronic medical records (EMRs). Apart from sociodemographic data of every patient, the EMR encompasses routinely registered data on contacts with the general practitioner. For this study the total number of face-to-face contacts with the GP in the year preceding the video consultation was included in the analyses as a measure for healthcare use.

Prior to the videotaped consultation patients completed a questionnaire in which they rated their overall health on 5-point Likert-type scales ('no limitation at all' to 'severely limited') [20] and familiarity to the GP they were consulting ('not at all/hardly', 'moderately', 'quite/very well').

#### GPs

Immediately after the consultation, GPs rated whether they believed psychological factors played a role in the health problem presented during the consultation on 5-point Likert-type scales (extremes labelled as 'not at all' and 'very much') and whether they had sufficient time for the consultation (no/yes). In addition, GPs rated how well they knew the patient ('not at all/hardly', 'moderately', 'quite/very well').

#### Familiarity

The score on the variable, 'knowing the doctor/patient well' was used as a proxy measure for personal continuity, comparable to other studies [7,9]. Combining the values on this variable of both the GP and the patient in a 3 × 3-table resulted in the construction of a new variable, to be referred to as '*familiarity*', with three categories. Sixty-three percent of the total sample of GP – patient couples (n = 2368) agreed on the degree of familiarity. The distribution over the three categories for the group who agreed was as follows: 'hardly or not at all familiar' (13.5%), 'moderately familiar' (17.8%) and 'very familiar' (68.7%).

### Data sample present study

#### Procedure of selection of GPs

Male and female GPs in the initial sample differed on a number of variables which might influence the hypotheses tested in the present study, such as age, number of years GPs are working in the present practice, number of weekly working hours, and number of patients per full time equivalent. Furthermore, there was a difference in rated familiarity with the patients between male and female GPs, with female GPs reporting to be familiar with more patients than their male colleagues. We could not exclude beforehand these characteristics to influence the variables studied. We wanted to guarantee that these variables were present in our sample in sufficient quantities to study the effects. In a random sample, female GPs might have been underrepresented as well as the characteristics mentioned. To include GP's gender in the analyses, every randomly chosen female GP (n = 20) was matched with a male GP (n = 20), regarding age, the number of years working in the present practice, number of working hours per week (expressed in full time equivalent: fte), practice type (single handed or not) and level of urbanization of the working area (city or countryside).

#### Procedure of selection of consultations: familiarity

The hypotheses were tested using a sample of 400 videotaped consultations, 10 of every GP. The selection of consultations was based on the degree to which physician and patient indicated to be familiar to each other, preferably couples who agreed on the level of familiarity. Because the number of consultations with a familiarity-score of 1 (not at all/hardly familiar) and 2 (moderately familiar) was smaller than category 3 (very familiar), consultations with familiarity scores 1 were chosen first, followed by consultations of the same GP scoring 2 and 3 on *familiarity*. We did not have enough GP-patient couples to fill the categories satisfactorily. Therefore we added some couples who did not agree fully. In these cases, we let the GP's opinion prevail. Ten percent of the couples in the resulting sample, did not agree fully, but disagreement was always minor (i.e. never more than one step on the scale).

From the sample, six videotaped consultations could not be linked to the GP- and patient-registration data due to missing patient-codes. Consequently, 394 patients/consultations were analyzed.

Table 1 shows the resulting group size and the characteristics of patients and GPs participating in this study.

**Table 1:** Sample characteristics

	Selected videotaped consultations
**Patients**	**N = 394**

Age	
-Range (years)	18–88
-Mean (SD)	49.4 (17.4)
Male (%)	40.6
Private health insurance (%)	26.1
Number of consultations	
-Range	1–58
-Mean (SD)	7.0 (6.6)
Self-reported overall health (%)	
-(very) good	56.5
-Moderate to poor	43.5
Familiarity (%):	
-hardly or not at all familiar	29.2
-moderately familiar	18.3
-very familiar	52.2

**GPs**	**N = 40**

Age	
-Range (years)	35–59
-Mean (SD)	45.9 (7.2)
Male (%)	50.0
Practicing years	
-Range	1–30
-Mean (SD)	14.4 (8.9)
Full time equivalent	
-Range	.20–1.00
-Mean (SD)	.80 (.20)
No. of patients per GP	
-Range	1345–2810
-Mean (SD)	2226 (362)
Duo-/group practice (%)	82.5
Location (%)	
-Most urban	17.5
-Urban	30.0
-Suburban	15.0
-Mixed urban/rural	25.0
-Rural	12.5

### Topics during consultation

Communication was rated by means of an observation checklist (table 2). Three topics of conversation were distinguished: (1) medical issues, (2) psychological themes, such as coping, stress, concerns, anxiety, and other emotions, and (3) the social environment of the patient such as partner, family, work, home situation. For each topic of conversation, a trained observer rated the following dimensions: (a) whether it received considerable attention or hardly/no attention, and (b) whether it was clear from the conversation that the GP had prior knowledge about the topic, which might indicate that the patient had visited the GP before with the same problem. This prior knowledge could be 'known by heart' or information that was readily available from the electronic medical record. We have chosen to combine these two 'modes of knowing' because it was not possible to differentiate between these two by observation of the consultation. The rating resulted in 6 variables (1a/b, 2a/b and 3a/b), which were introduced as dependent variables in the analyses.

**Table 2:** What were the topics of the conversation? (N = 394 consultations)

	Yes (%)
**Medical issues **received considerable attention during consultation	88.1
During these consultations (n = 347) GP displayed prior knowledge	51.0
	
**Psychological themes **received considerable attention during consultation	39.3
During these consultations (n = 154) GP displayed prior knowledge	41.3
	
**Social environment **of patient received considerable attention during consultation	38.6
During these consultations (n = 151) GP displayed prior knowledge	53.6

All consultations were evaluated by two experienced observers. They were blind with respect to individual characteristics of the GP-patient dyad. To assess interrater reliability, 20 consultations were rated by the two observers who were involved. Items from the checklist had good inter-observer reliability as indicated by a mean Spearman correlation of 0.83 (range 0.63–1.0).

### Analyses

The hierarchical structure of the data was taken into account by applying multilevel logistic regression analysis with the topics of conversation as the dependent variables. Multilevel analysis corrects for clustering of data, when patients are sampled (nested) within GPs. The intraclass correlations in our 'empty' models ranged between 0.00 and 0.115, which indicated that for some dependent variables a small part of the variance could be explained by differences between GPs.

The explanatory variable 'familiarity' was included in the models with two dummy variables for 'moderately familiar' and 'very familiar', with 'not familiar' as reference category. GP and patient characteristics were studied as potentially explaining factors. Because some of the explanatory variables were correlated, we checked for multicollinearity. This was not found to be a problem.

### Ethical approval

The study was carried out according to Dutch legislation on privacy. The privacy regulation of the study was approved by the Dutch Data Protection Authority.

## Results and discussion

### Topics of conversation

Medical issues rank number 1 on the list of topics of the conversation (table 2). In more than half of these consultations GPs displayed prior knowledge suggesting that there had been communication before about this problem.

In about forty percent of all consultations, psychological themes and/or the social environment of the patient received considerable attention (table 2). In about half of these consultations the GP displayed prior knowledge, suggesting that these topics had been discussed before. These results show that the GP plays a role in psychosocial care of his/her patients. Apparently, patients and GPs feel free to discuss the same topics again.

### Variables predicting conversation topics

Subsequently we analyzed which patient and GP characteristics could explain differences in conversation topics. Table 3 presents significant associations (expressed as odds ratios) between patient and GP characteristics on the one hand, and topics of the conversation on the other.

**Table 3:** Associations between GP-/patient-characteristics and contents of the consultation*

Dependent variables	N	Predictor	OR (95% CI)
**Medical issues**			
a. Received considerable attention	394	Bad overall health	1.40 (1.01–1.95)
		Psychological factors	0.70 (0.52–0.93)
b. The GP displayed prior knowledge	347	**Very familiar†**	**3.58 (1.76–7.25)**
		Duo/group practice	3.16 (1.27–7.86)
			
**Psychological themes**			
a. Received considerable attention	394	Psychological factors	1.96 (1.59–2.43)
b. The GP displayed prior knowledge	154	**Very familiar†**	**13.57 (2.52–73.23)**
		Bad overall health	0.64 (0.42–0.97)
		Psychological factors	1.66 (1.15–2.41)
			
**Social environment**			
a. Received considerable attention	394	Psychological factors	1.64 (1.33–2.01)
b. The GP displayed prior knowledge	151	**Moderately familiar†**	**6.49 (1.39–30.28)**
		**Very familiar†**	**8.07 (2.22–29.36)**

*only significant results are shown (p < 0.05)

**† **reference category is 'not or hardly familiar with each other'

Variables entered in the model: (a) GP and patient moderately familiar or (b) GP and patient very familiar, (c) overall health as rated by the patient, (d) role of psychological factors in the complaint as perceived by the GP, (e) GP's sex, (f) number of working hours per week (fte), (g) the number of patients per full time equivalent, (h) number of years GPs were practicing in the present practice, (i) sufficient time for the consultation, (j) working in a duo-/group-practice, (k) degree of urbanisation, (l) patient's sex, (m) patient's age, (n) number of consultations one year preceding the video-consultation.

#### Medical issues

Medical issues were more often discussed when patients reported worse overall health and received less attention when GPs thought that health problems had a psychological background. The chance of the GP displaying prior knowledge on medical issues was increased when GP and patient were very familiar with each other. GPs operating in a duo or group practice displayed prior knowledge more often than colleagues working single-handed. The reason for this might be that in duo and group practices the need to record specific details of consultations is higher than in single-handed practices, because next time the patient might be visiting a colleague. Probably, 'prior knowledge' finds its source here in well documented electronic medical records, read by the GP before the patient enters the consultation room.

#### Psychological themes and the social environment

Psychological themes and the social environment were discussed more often when the GP thought that psychological factors played a role in the health problem. GPs completed the questionnaire after the consultation; therefore, it is not surprising that when psychological themes had been discussed the GP indicated that psychological factors might play a role. Familiarity was not found to add explanatory power. However, when the GP and the patient were familiar with each other, GPs also displayed more often prior knowledge on psychological themes and the social environment of the patient. Worse overall health of the patient was associated with the GP displaying less prior knowledge on psychological themes.

In addition to the variables mentioned above, we have entered more variables into the equation that might have influenced the communication. Gender and age of GP and patient are known to influence communication patterns [21,22]. Instead of controlling for age, we used the number of years the GP worked in the present practice, because this might influence the intensity of the relationship with patients [23]. A GP who has 'lived' for thirty years with his patients in the same practice and who knows their families and their background, might communicate differently with his patients [24]. The effect of degree of urbanization might also play a role, with patients being geographically less mobile in rural areas, which might influence the duration of the relationship and familiarity between GP and patient [25]. However, no effects of any of these variables were found in this study. Having enough time for the consultation might give GPs the opportunity for more extensive communication. But we did not find any relationship between communication patterns and (a) the number of patients per full time equivalent per GP (objective measure for work load), (b) number of working hours a week or (c) the subjective judgment of the GP on 'having sufficient time for this patient during the consultation'.

We carefully conclude that the relationship between familiarity and the content of the conversation is independent of these potentially biasing factors.

### Limitations of the study

Some methodological remarks should be made with respect to these findings. We found wide confidence intervals for some of the variables in the regression analyses, which were probably caused by the relatively small sample size. This means that results have to be interpreted with some care. Also, we could not distinguish between consultations for serious conditions and those for minor complaints. This is a relevant contrast in research on continuity of care, as personal continuity is more often valued for more serious problems [15,16].

We combined GP and patient ratings on their familiarity with each other into one measure. Due to a limited number of GP-patient couples, we also had to include consultations in which GPs and patients did not fully agree on the degree of familiarity. Because it is the GP with the information gap (or information need) and he/she is generally guiding the conversation we decided to let his/her opinion prevail. Ten percent of the couples did not agree fully, but disagreement was always minor (i.e. never more than one step on the scale). Therefore, we do not think this has influenced the results to a great extent.

### Considerable attention

We rated whether topics received considerable attention during the consultation and did not rate topics receiving only slight attention. The reason is that we wanted to have a measure as robust as possible and even then interpretation is complex. What does it mean when a topic is discussed? We rated only one consultation with the patient and as a consequence we do not know which topics have been discussed during prior consultations. Does the patient bring it up for the first time now? Or does the patient tell this story repetitively? Does it relieve the patient or is it painful [26]? Are patient and GP so familiar that half a word or one glance is sufficient to understand each other? Every patient is different, as is every GP. And on top of it, the interaction between a patient and a GP might have resulted in a different communication pattern with another (familiar) GP. We could not distinguish any of these nuances because we observed only one consultation per patient. But the observation we could make was that familiarity did not appear to lead to different topics.

### Familiarity and personal continuity

Continuity is a broad concept with different dimensions and definitions. In general, practice continuity of care is almost synonymous with care by one doctor, usually over an extended time span and concerning more than one episode of illness [27]. Different aspects of continuity of care are combined in this definition, such as informational continuity which is defined as the availability of accurate information from one health care encounter to another; longitudinal continuity of care delivered during a longer period by the same person or by as few persons as possible; and personal continuity defined as a personal doctor-patient relationship in which the patient can establish and maintain a therapeutic relationship which is characterized by loyalty and trust [1,27,28]. Continuity of care has also been studied by defining it as 'knowing patients, knowing about patients' [17] or 'accumulated knowledge about patients' [2,3]. In this study we used 'familiarity' operationalized as 'knowing the patient well' and 'knowing the GP well' as a proxy for personal continuity [9].

It is remarkable that in our study familiarity did not influence the topics brought up during the consultation. This might be less surprising for medical issues, but was not expected for the non-medical issues. We expected a lower threshold for psychological themes such as emotions, anxieties, stress or coping and topics relating to the patient's social environment, such as partner, family or work. Independent of familiarity, GPs seem to be very well capable of putting the patient at ease and giving the opportunity to discuss all topics in the consultation, including non-medical issues. Or as Tarrant et al. state 'Personal care is promoted by but not always dependent on a continuing provider-patient relationship [13].

Communication has always been an important theme in patients' experience of personal continuity. In fact, good communication might just be what patients are looking for when they ask for a personal doctor, because continuity with a physician leads to increased knowledge and trust between a patient and a physician [11,13,29]. Tarrant and colleagues found that patients who have emotional problems are often prepared to wait for an appointment with a GP who is familiar with their background and concerns [13]. Not only patients but also GPs still place high value on personal continuity of care for their patients [13,30].

Nevertheless, the traditional long-lasting relationship between GPs and patients has been put under increasing pressure. Practices are expanding, with larger teams and more enlisted patients, thereby threatening the possibility for patients to choose their doctor and stay with him. A decrease of personal continuity has been argued to increase the importance of informational continuity. This implies that providers should have access to comprehensive information about the patient if different providers in different locations provide the care [31]. Informational continuity is mainly concerned with the registration of medical information and recordkeeping [32]. In this study we found indications for well organized informational continuity: GPs working in group practices showed prior knowledge of medical problems brought up by the patient.

However, knowing a medical history is not the same as knowing a patient [31,33]. Especially for patients experiencing psychological problems, a medical history is not the only history that is relevant. Knowledge about the patient's preferences, values, and context is usually gathered over time, accumulating in the memory of providers who interact with the patient [33]. The contribution of this study is the finding that communication skills of GPs, whether they are familiar with the patient or not, enable patients to discuss any issue, thereby guaranteeing 'whole person care'.

## Conclusion

Not unexpectedly, when GPs and patients are familiar with each other, GPs more often display prior knowledge on medical issues, psychological themes and social environmental aspects brought up during the consultation.

Our results suggest that familiarity between GP and patient was not related to the content of the consultation. This is remarkable because we expected that familiarity would 'open up the communication' for more psychological and social themes. GPs seem to have the communication skills to enable patients to discuss any issue, no matter how familiar GP and patient are with each other.

## Competing interests

AT and GAvE are both connected to the Dutch College of General Practitioners which funded this project: AT is director, GAvE advisor.

## Authors' contributions

FS, AT and GAvE formulated the research question. LJ, TF, FS and SvD participated in the design of the study. TF performed the statistical analyses and drafted the manuscript. LJ wrote the final version of the manuscript. SvD and FS supervised the study. All authors have read and approved the final manuscript.

## Pre-publication history

The pre-publication history for this paper can be accessed here:


